# Dynamics of Neutrophil-to-Lymphocyte Ratio (NLR), Lymphocyte-to-Monocyte Ratio (LMR), and Platelet-to-Lymphocyte Ratio (PLR) in Patients with Deep Neck Infection

**DOI:** 10.3390/jcm13206105

**Published:** 2024-10-13

**Authors:** Jeong-Mi Kim, Huu Hoang, Jeong-Seok Choi

**Affiliations:** 1 Department of Otorhinolaryngology-Head and Neck Surgery, College of Medicine, Inha University, 27 Inhang-ro, Incheon 22332, Republic of Korea; 316417@inha.ac.kr (J.-M.K.); hhuu@huemed-univ.edu.vn (H.H.); 2 Research Center for Controlling Intercellular Communication (RCIC), College of Medicine, Inha University, 100 Inha-ro, Incheon 22212, Republic of Korea; 3 Department of Oncology, Hue University of Medicine and Pharmacy, 06 Ngo Quyen Street, Vinh Ninh Ward, Hue City 49000, Vietnam; 4 Department of Biomedical Science, Program in Biomedical Science & Engineering, Inha University, 100 Inha-ro, Incheon 22212, Republic of Korea

**Keywords:** deep neck infection, inflammatory biomarkers, neutrophil-to-lymphocyte ratio (NLR), platelet-to-lymphocyte ratio (PLR), lymphocyte-to-monocyte ratio (LMR)

## Abstract

**Background:** Inflammatory biomarkers, including the neutrophil-to-lymphocyte ratio (NLR), lymphocyte-to-monocyte ratio (LMR), and platelet-to-lymphocyte ratio (PLR), have been utilized as prognostic factors in various diseases. This study aims to evaluate changes in the NLR, PLR, and LMR in patients diagnosed with a deep neck infections (DNI) to identify useful prognostic markers. **Methods:** This single-center, retrospective cohort study utilized data from the electronic medical records of patients admitted to the ENT department of a tertiary university hospital between January 2000 and August 2024. Patients diagnosed with a DNI during the study period were enrolled. Preoperative and postoperative inflammatory markers were measured in all patients, and NLR, LMR, and PLR values were calculated and analyzed. **Results:** The post-treatment NLR was significantly lower than the pre-treatment NLR. Similarly, the post-treatment LMR was significantly higher and the post-treatment PLR was significantly lower compared to pre-treatment values. Patients admitted to the ICU had higher inflammatory markers than those in general wards. Additionally, patients with elevated inflammatory markers had longer hospital stays. Inflammatory markers were also higher in older patients and those who underwent surgical treatment. **Conclusions:** Significant changes in the NLR, LMR, and PLR in patients diagnosed with a DNI can serve as useful prognostic markers. These findings suggest that monitoring these markers may help to assess and improve the inflammatory status of patients, highlighting their potential role in guiding treatment.

## 1. Introduction

Deep neck infections (DNIs) are rapidly progressing conditions that affect the deep cervical spaces or fascial planes of the neck and are relatively common [1]. They are associated with serious and potentially life-threatening complications, including mediastinitis, septic thrombophlebitis, pericarditis, and airway obstruction [2]. While odontogenic infections are the most frequent cause of DNIs, other causes include acute pharyngeal infections, cervicofacial trauma, bacterial infections of branchial arch remnants, and tuberculosis [3]. Various clinical and serological factors have been studied to assess the severity, prognosis, complications, need for surgical debridement, and mortality in patients with a DNI. Inflammatory biomarkers, such as C-reactive protein (CRP), the erythrocyte sedimentation rate (ESR), and the systemic immune inflammation index (SII), have shown prognostic value in sepsis and infections, but their specific role in DNIs remains unclear [4,5,6,7,8].

In recent years, hematologic inflammatory markers have gained increasing attention as potential indicators of systemic inflammation and disease severity in various clinical settings [9]. Among these markers, the neutrophil-to-lymphocyte ratio (NLR), lymphocyte-to-monocyte ratio (LMR), and platelet-to-lymphocyte ratio (PLR) have been particularly studied. These ratios, derived from routine complete blood counts (CBCs), are convenient tools for assessing patients’ inflammatory status.

The NLR, calculated by dividing the neutrophil count by the lymphocyte count, has been widely studied as a marker of inflammation and stress response in various conditions, including cardiovascular diseases, infections, and cancers [10,11]. Elevated NLR levels have been associated with poorer outcomes in numerous clinical settings, reflecting heightened inflammation and immune dysregulation. Similarly, the LMR, which is calculated by dividing the lymphocyte count by the monocyte count, has been investigated as a prognostic marker in malignancies and inflammatory diseases [12,13], with lower LMR values suggesting a more severe inflammatory response. The PLR, calculated by dividing the platelet count by the lymphocyte count, has also been explored as an inflammatory marker, especially in cardiovascular and oncological contexts, where higher PLR values often correlate with worse clinical outcomes.

Research on the relationship between DNI and systemic inflammation, as measured by the NLR, LMR, and PLR, is limited. Understanding how these markers change in response to treatment could offer valuable insights into the inflammatory processes involved and reveal the potential benefits of treatment beyond symptom relief. This study aims to enhance our understanding of the inflammatory dynamics associated with a DNI. Identifying significant changes in the NLR, LMR, and PLR may also help in surgical and medical management, providing clinicians with additional tools for assessing a patient’s status and prognosis.

## 2. Materials and Methods

### 2.1. Study Design and Population

This study, approved by the Inha University Hospital Ethics Committee, included 965 patients diagnosed with a DNI who were admitted to the Department of Otolaryngology between January 2000 and August 2024. A DNI was confirmed by the presence of abscesses identified through ring enhancement on imaging studies, such as computed tomography (CT). Exclusion criteria included (1) patients with abscesses caused by foreign bodies, (2) those diagnosed with head, oral, or neck diseases such as cancer, and (3) patients with compromised immune systems. All patients with a DNI received antibiotic therapy, and corticosteroids were administered in cases where airway compromise was a concern. Of the 965 patients, 288 (29.84%) underwent surgery, with 237 receiving debridement and drainage, 10 undergoing thoracostomy, and 41 receiving a tracheostomy. Hematologic analyses were based on blood tests conducted at the time of admission and prior to discharge.

### 2.2. Data Collection

Patient data were collected from electronic medical records (EMRs), including demographics (age and gender), laboratory findings (white blood cell [WBC], neutrophil, monocyte, lymphocyte, and platelet counts), the presence of diabetes, treatment outcomes (discharge, general ward [GW], or ICU admission, and surgical procedure), and the length of hospital stay. For all patients, preoperative and postoperative blood cell counts were obtained and compared. Patients who lacked postoperative blood counts or were lost to follow-up were excluded.

### 2.3. Statistics

Normality was assessed using the Shapiro–Wilk test. For comparisons of quantitative variables and non-normally distributed parameters between groups, the Wilcoxon rank-sum test, chi-square test, and Kruskal–Wallis test were used, with Dunn’s test for post-hoc analysis when appropriate. Data were presented as the median [interquartile range, IQR]. All statistical analyses were performed using GraphPad Prism software Version 8, with significance set at *p* < 0.05.

## 3. Results

### 3.1. Demographic and Clinical Data

A total of 965 patients diagnosed with a DNI were included. The mean age was 48.41 ± 19.69 years, with 596 males (61.76%) and 369 females (38.24%). There was no significant gender-based difference in DNI incidence. Diabetes mellitus was present in 3.83% (n = 37) of cases. Of the patients, 29.84% underwent surgery, and 11.09% were admitted to the ICU. The average ICU stay was 120.88 ± 210.66 h, and the average length of stay in the GW was 12.01 ± 17.92 days (Table 1).

### 3.2. Hematologic Analysis under Various Comparative Conditions

An analysis of blood cell counts by gender revealed that, prior to treatment, males had higher WBC and monocyte counts compared to females, while their lymphocyte and platelet counts were lower. After treatment, males continued to exhibit higher WBC and monocyte counts, as well as decreased platelet counts compared to females. However, no significant differences were observed in neutrophil or lymphocyte counts between genders. In both males and females, WBC and neutrophil counts decreased post-treatment compared to pre-treatment, while monocyte and lymphocyte counts increased (Table 2, *p* < 0.05).

Patients were divided into two groups based on an age cutoff of 60 years, and differences in blood cell counts between these groups were compared. In patients aged 60 years and above, neutrophil levels were higher, while lymphocyte, monocyte, and platelet levels were lower compared to those under 60 years. This pattern persisted post-treatment. In both age groups, WBC and neutrophil counts decreased post-treatment compared to pre-treatment, while lymphocyte and monocyte counts increased (Table 3, *p* < 0.05).

When comparing blood cell counts between patients with and without diabetes, no significant differences were observed between the two groups before treatment. However, in both groups, WBC and neutrophil counts significantly decreased post-treatment compared to pre-treatment, while lymphocyte counts significantly increased. Monocyte counts significantly increased post-treatment only in patients without diabetes (Table 4, *p* < 0.05).

A comparison of blood cell counts based on surgical status showed that patients who did not undergo surgery had lower WBC and neutrophil levels but higher lymphocyte and monocyte levels compared to those who underwent surgery. In both groups, WBC and neutrophil levels decreased post-treatment compared to pre-treatment, while lymphocyte and monocyte levels increased (Table 5, *p* < 0.05).

Patients admitted to the ICU had higher WBC and neutrophil counts and lower lymphocyte and monocyte counts compared to those admitted to the general ward (GW), a trend that persisted post-treatment. In GW patients, WBC and neutrophil counts decreased post-treatment compared to pre-treatment, while lymphocyte and monocyte counts increased. In contrast, ICU patients experienced decreases in WBC, monocyte, and platelet counts post-treatment compared to pre-treatment (Table 6, *p* < 0.05).

We also divided patients into two groups based on a 15-day hospitalization period to compare blood cell counts between long-term and short-to-mid-term hospitalizations. Patients hospitalized for more than 15 days had lower WBC and neutrophil counts and higher lymphocyte and monocyte counts compared to those hospitalized for less than 15 days. Post-treatment, patients in both groups exhibited lower WBC and neutrophil counts, and higher lymphocyte and monocyte counts compared to pre-treatment. Additionally, those hospitalized for more than 15 days had lower post-treatment WBC and neutrophil counts and higher lymphocyte and monocyte counts compared to those hospitalized for less than 15 days (Table 7, *p* < 0.05).

### 3.3. Variations in NLR, PLR, and LMR Based on Gender, Age, Diabetes, and Pre/Post-Treatment Intervals

**Figure 1:**
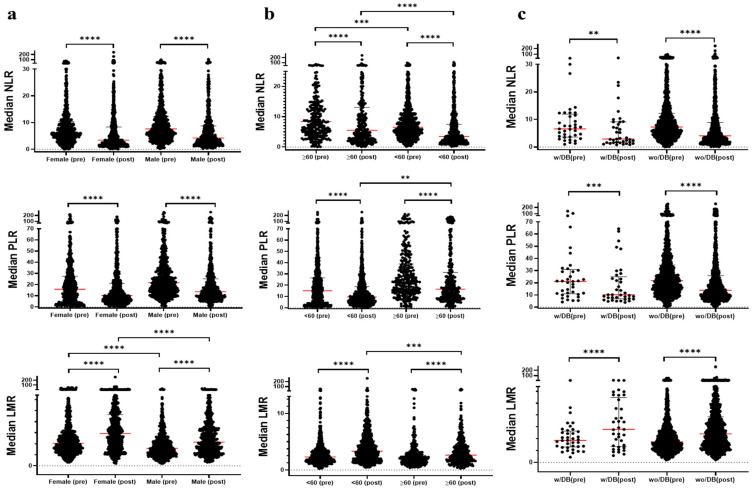
A comparison of NLR, PLR, and LMR values according to (**a**) gender, (**b**) age and (**c**) diabetes (DB), and pre-and post-treatment periods.

The LMR was significantly lower in males compared to females, while the NLR and PLR showed no significant gender differences. Post-treatment, the NLR and PLR decreased, while the LMR increased in both genders. The NLR was higher in patients aged 60 and above, with the post-treatment NLR and PLR remaining higher and the LMR lower compared to younger patients (Figure 1, *p* < 0.05).

The pre-treatment (pre-) and post-treatment (post-) values of the neutrophil-to-lymphocyte ratio (NLR), lymphocyte-to-monocyte ratio (LMR), and platelet-to-lymphocyte ratio (PLR) across three groups of gender, age, and diabetes status are presented. Values are shown as the median [IQR]. Statistical significance between pre- and post-values was assessed using the Wilcoxon matched-pairs signed rank test. *p* indicates the statistical significance between the two indicated groups; ** *p* < 0.01, *** *p* < 0.001, **** *p* < 0.0001.

### 3.4. Changes in the NLR, PLR, and LMR Based on Surgery, ICU Admission, and Length of Hospital Stay

**Figure 2:**
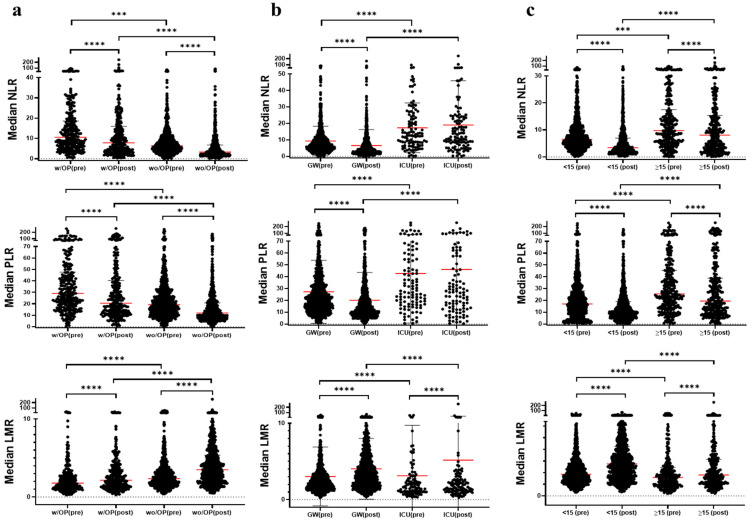
A comparison of NLR, PLR and LMR values according to (**a**) surgery status, (**b**) ICU admission, and (**c**) length of hospital stay, and pre-and post-treatment periods.

Patients undergoing surgery or admitted to the ICU had higher NLR and PLR values and lower LMR values compared to non-surgical or GW patients. These markers changed post-treatment, reflecting improvements in systemic inflammation (Figure 2, *p* < 0.05).

The pre-treatment (pre-) and post-treatment (post-) values of the neutrophil-to-lymphocyte ratio (NLR), lymphocyte-to-monocyte ratio (LMR), and platelet-to-lymphocyte ratio (PLR) across the three groups of surgery status, ICU admission, and length of hospital stay are presented. Values are shown as the median [IQR]. Statistical significance between pre- and post-values was assessed using the Wilcoxon matched-pairs signed rank test. *p* indicates the statistical significance between the two indicated groups; *** *p* < 0.001, **** *p* < 0.0001.

## 4. Discussion

This study highlights the prognostic value of the NLR, LMR, and PLR in assessing the severity and progression of a DNI. Our findings indicate that significant changes in these markers post-treatment correspond with a reduction in systemic inflammation and improved patient outcomes.

A comparison of the NLR, PLR, and LMR between male and female patients with a DNI revealed that the pre-treatment LMR levels were higher in females than in males, with no significant differences observed in the NLR and PLR levels. This trend remained consistent post-treatment. According to the literature, females tend to have higher LMR levels than males in healthy populations, while NLR and PLR patterns may vary slightly depending on the study [14,15,16]. The higher LMR levels in females compared to males with a DNI are thought to reflect inherent gender differences rather than disease-related factors.

Our study shows that the post-treatment NLR is significantly lower than the pre-treatment values, while the post-treatment LMR is significantly higher, and the post-treatment PLR is significantly lower. These changes suggest an improvement in the systemic inflammatory response following treatment. An elevated NLR is often associated with increased inflammation and immune dysregulation, which reflect worse prognoses in various clinical settings. The reduction in the NLR after treatment suggests a decrease in systemic inflammation, which corresponds with the clinical improvement of patients with a DNI [17,18,19,20,21]. Similarly, the increase in the LMR and decrease in the PLR post-treatment further support the idea that treatment effectively modulates systemic inflammation. A higher LMR typically indicates a more balanced inflammatory response, while a lower PLR can reflect reduced platelet activation and inflammation [22].

Our study also highlights that patients admitted to the intensive care unit (ICU) had higher inflammatory markers compared to those admitted to general wards (GWs). This finding underscores the severity of DNIs in ICU patients, who typically present with more complex or advanced disease. Additionally, patients with higher inflammatory markers tended to have a longer length of hospital stay, aligning with the established correlation between elevated inflammation and prolonged recovery times [23,24].

Inflammatory markers were notably higher in older patients and those undergoing surgical treatment. This may be attributed to older individuals having a more pronounced inflammatory response or underlying comorbidities that exacerbate inflammation. While necessary, surgical procedures also induce a systemic inflammatory response, which could contribute to the observed elevations in these inflammatory markers.

The identification of significant changes in the NLR, LMR, and PLR provides promising prognostic insights for patients with a DNI. Monitoring these markers can help clinicians assess the severity of the infection, predict treatment outcomes, and plan appropriate management strategies. For example, a persistently high NLR or low LMR post-treatment may indicate ongoing inflammation or complications, prompting further intervention.

The practical application of these markers is further supported by their ease of calculation from routine complete blood counts (CBCs). This makes them accessible tools for clinicians to evaluate patients’ inflammatory status and adjust treatment plans accordingly.

Despite the valuable insights offered by this study, limitations include its retrospective design and reliance on electronic medical records (EMRs), which may introduce bias. Future research should explore additional inflammatory markers and include larger, more diverse patient populations to validate these findings further.

## 5. Conclusions

The NLR, LMR, and PLR are useful indicators of systemic inflammation and disease severity in patients with a DNI. Changes in these markers post-treatment offer valuable prognostic insights and may aid clinicians in monitoring patient progress and optimizing care. Further research is needed to establish definitive thresholds for these markers in DNIs and integrate them into routine clinical practice.

## Figures and Tables

**Table 1:** Demographic and characteristics of patients.

Variable		Deep Neck Infection (n = 965)
Gender	Male	596 (61.76%)
	Female	369 (38.24%)
Age		48.41 ± 19.69
Diabetes Mellitus		37 (3.83%)
Underwent Surgery		288 (29.84%)
Hospitalization Period (days)		12.01 ± 17.92
Intensive-Care-Unit Stay (hours)		120.88 ± 210.66

The hematological characteristics of patients with deep neck infections. Data presented as mean ± SD. The gender differences in patients with a DNI were analyzed using the chi-square test.

**Table 2:** Hematological parameters and NLR, LMR, and PLR values according to gender.

Variable	Gender							
	Female					Male		
	Pre-Treatment	Post-Treatment	*p*	p *	*p ^#^*	Pre-Treatment	Post-Treatment	*p*
WBCs (×10^3^/μL)	12.32 [6.66]	7.86 [6.67]	<0.0001	<0.0001	0.0223	14.11 [7.15]	8.80 [6.24]	<0.0001
Neutrophils (× 10^3^/μL)	81.20 [13.80]	70.90 [27.20]	<0.0001	0.4943	0.5942	80.25 [11.50]	72.20 [22.07]	<0.0001
Lymphocytes (× 10^3^/μL)	11.80 [10.45]	18.80 [22.25]	<0.0001	0.0360	0.1014	10.70 [9.10]	17.00 [17.10]	<0.0001
Monocytes (× 10^3^/μL)	4.40 [2.30]	5.00 [2.70]	0.0002	<0.0001	<0.0001	5.40 [2.80]	5.90 [2.60]	<0.0001
Platelets (× 10^3^/μL)	246.00 [109.50]	248.00 [120.00]	0.1762	0.0051	0.0028	231.00 [90.00]	234.00 [104.50]	0.0580
NLR	7.01 [7.90]	3.70 [7.67]	<0.0001	0.0854	0.1720	7.60 [7.71]	4.23 [6.57]	<0.0001
LMR	2.57 [2.09]	3.5 [3.75]	<0.0001	<0.0001	<0.0001	1.98 [1.62]	2.69 [2.67]	<0.0001
PLR	21.58 [22.67]	14.14 [18.92]	<0.0001	0.7190	0.6818	21.63 [19.90].	13.55 [16.41]	<0.0001

The hematological parameters, including WBCs, neutrophils, lymphocytes, monocytes, platelets, and their derived ratios (NLR, LMR, PLR) before (pre-) and after (post-) treatment for patients categorized by gender are presented. Data are presented as median [IQR]. The *p*-values were calculated using the Wilcoxon matched-pairs signed rank test for within-group comparisons. *p* indicates the comparison between pre- and post-treatment within each group; *p* * compares pre-treatment values between the two groups; and *p* ^#^ compares post-treatment values between the two groups.

**Table 3:** Hematological parameters and NLR, LMR, and PLR values according to age.

Variable	Age							
	<60					≥60		
	Pre-Treatment	Post-Treatment	*p*	p *	*p ^#^*	Pre-Treatment	Post-Treatment	*p*
WBCs (× 10^3^/μL)	13.46 [6.85]	8.32 [5.91]	<0.0001	0.0527	0.0529	12.50 [7.81]	9.17 [7.71]	<0.0001
Neutrophils (× 10^3^/μL)	79.95 [11.57]	70.15 [23.77]	<0.0001	<0.0001	<0.0001	82.90 [12.85]	76.50 [24.60]	<0.0001
Lymphocytes (× 10^3^/μL)	11.70 [9.30]	19.50 [19.48]	<0.0001	0.0003	<0.0001	9.80 [9.65]	13.70 [17.65]	<0.0001
Monocytes (× 10^3^/μL)	5.20 [2.7]	5.75 [2.68]	<0.0001	<0.0001	<0.0001	4.60 [2.55]	5.10 [3.10]	0.0082
Platelets (× 10^3^/μL)	240.00 [97.80]	247.00 [105.70]	0.0022	0.0046	0.0002	222.00 [110.00]	222.00 [116.00]	0.6198
NLR	6.88 [6.55]	3.60 [5.86]	<0.0001	0.0003	<0.0001	8.41 [10.22]	5.51 [10.58]	<0.0001
LMR	2.23 [1.82]	3.21 [3.13]	<0.0001	0.0968	0.0004	2.07 [1.77]	2.64 [2.68]	<0.0001
PLR	20.90 [19.28]	12.83 [15.15]	<0.0001	0.0559	0.0020	23.00 [25.10]	16.46 [22.57]	<0.0001

The hematological parameters, including WBCs, neutrophils, lymphocytes, monocytes, platelets, and their derived ratios (NLR, LMR, PLR) before (pre-) and after (post-) treatment for patients categorized by age are presented. Data are presented as median [IQR]. The *p*-values were calculated using the Wilcoxon matched-pairs signed rank test for within-group comparisons. *p* indicates the comparison between pre- and post-treatment within each group; *p* * compares pre-treatment values between the two groups; and *p* ^#^ compares post-treatment values between the two groups.

**Table 4:** Hematological parameters and NLR, LMR, and PLR values of patients according to their diabetes status.

Variable	Diabetes							
	With					Without		
	Pre-Treatment	Post-Treatment	*p*	p *	*p ^#^*	Pre-Treatment	Post-Treatment	*p*
WBCs (× 10^3^/μL)	11.52 [7.61]	7.90 [4.74]	<0.0001	0.2530	0.3591	13.25 [7.16]	8.55 [6.50]	<0.0001
Neutrophils (× 10^3^/μL)	79.20 [12.50]	67.70 [25.90]	<0.0001	0.2004	0.2526	80.70 [11.90]	72.00 [24.27]	<0.0001
Lymphocytes (× 10^3^/μL)	12.10 [13.15]	22.80 [25.80]	<0.0001	0.3206	0.2834	11.15 [9.58]	17.70 [18.90]	<0.0001
Monocytes (× 10^3^/μL)	5.70 [2.35]	5.20 [2.95]	0.5936	0.2668	0.9550	5.00 [2.80]	5.50 [3.00]	<0.0001
Platelets (× 10^3^/μL)	223.00 [120.50]	235.00 [106.00]	0.3635	0.7340	0.4001	237.00 [101.00]	240.00 [113.00]	0.0106
NLR	6.57 [8.33]	2.91 [7.54]	0.0059	0.3181	0.3092	7.25 [7.79]	4.05 [6.89]	<0.0001
LMR	2.33 [1.69]	3.48 [5.16]	<0.0001	0.6150	0.3224	2.17 [1.84]	3.01 [3.02]	<0.0001
PLR	21.11 [20.10]	10.16 [18.02]	0.0003	0.3098	0.2175	21.72 [20.95]	13.86 [17.34]	<0.0001

The hematological parameters, including WBCs, neutrophils, lymphocytes, monocytes, platelets, and their derived ratios (NLR, LMR, PLR) before (pre-) and after (post-) treatment for patients categorized by diabetes status are presented. Data are presented as median [IQR]. The *p*-values were calculated using the Wilcoxon matched-pairs signed rank test for within-group comparisons. *p* indicates the comparison between pre- and post-treatment within each group; *p* * compares pre-treatment values between the two groups; and *p* ^#^ compares post-treatment values between the two groups.

**Table 5:** Hematological parameters and NLR, LMR, and PLR values of patient with/without under-goingsurgery.

Variable	Surgery							
	With					Without		
	Pre-Treatment	Post-Treatment	*p*	p*	*p ^#^*	Pre-Treatment	Post-Treatment	*p*
WBCs (× 10^3^/μL)	14.89 [9.18]	11.01 [9.11]	<0.0001	<0.0001	<0.0001	12.67 [6.59]	7.74 [4.99]	<0.0001
Neutrophils (× 10^3^/μL)	84.20 [11.37]	80.80 [18.58]	<0.0001	<0.0001	<0.0001	79.40 [12.45]	69.00 [23.85]	<0.0001
Lymphocytes (× 10^3^/μL)	8.05 [8.38]	10.30 [13.55]	<0.0001	<0.0001	<0.0001	12.60 [9.30]	21.00 [19.98]	<0.0001
Monocytes (× 10^3^/μL)	4.50 [2.40]	4.90 [3.30]	0.0308	<0.0001	<0.0001	5.30 [2.75]	5.80 [2.55]	<0.0001
Platelets (× 10^3^/μL)	239.00 [126.70]	236.50 [141.00]	0.3393	0.2109	0.9028	235.00 [90.00]	240.00 [104.00]	0.0006
NLR	6.38 [5.89]	3.29 [4.98]	<0.0001	0.0002	<0.0001	7.28 [7.94]	3.98 [6.92]	<0.0001
LMR	1.78 [1.62]	2.15 [2.22]	<0.0001	<0.0001	<0.0001	2.35 [1.81]	3.49 [3.27]	<0.0001
PLR	29.11 [29.63]	20.53 [28.35]	<0.0001	<0.0001	<0.0001	19.27 [17.57]	12.00 [13.16]	<0.0001

The hematological parameters, including WBCs, neutrophils, lymphocytes, monocytes, platelets, and their derived ratios (NLR, LMR, PLR) before (pre-) and after (post-) treatment for patients categorized by surgery are presented. Data are presented as median [IQR]. The *p*-values were calculated using the Wilcoxon matched-pairs signed rank test for within-group comparisons. *p* indicates the comparison between pre- and post-treatment within each group; *p* * compares pre-treatment values between the two groups; and *p* ^#^ compares post-treatment values between the two groups.

**Table 6:** Hematological parameters and NLR, LMR, and PLR values of patients according to ICU admission.

Variable	Group							
	GW					ICU		
	Pre-Treatment	Post-Treatment	*p*	p *	*p ^#^*	Pre-Treatment	Post-Treatment	*p*
WBCs (× 10^3^/μL)	12.94 [7.00]	8.18 [5.81]	<0.0001	0.0033	<0.0001	15.20 [9.63]	12.75 [10.73]	0.0007
Neutrophils (× 10^3^/μL)	79.90 [12.02]	70.10 [23.65]	<0.0001	<0.0001	<0.0001	85.00 [10.10]	86.30 [11.40]	0.7794
Lymphocytes (× 10^3^/μL)	11.70 [9.52]	19.05 [19.25]	<0.0001	<0.0001	<0.0001	6.50 [8.00]	7.30 [7.60]	0.5901
Monocytes (× 10^3^/μL)	5.10 [2.60]	5.80 [2.70]	0.0308	<0.0001	<0.0001	4.20 [2.40]	3.80 [2.80]	<0.0001
Platelets (× 10^3^/μL)	237.00 [99.00]	243.00 [107.30]	0.3393	0.2109	0.9028	217.00 [136.00]	195.00 [119.00]	0.0006
NLR	6.82 [6.78]	3.60 [5.67]	<0.0001	<0.0001	<0.0001	12.57 [17.02]	11.81 [16.50]	0.7271
LMR	2.24 [1.80]	3.22 [3.06]	<0.0001	<0.0001	<0.0001	1.63 [1.73]	1.65 [1.58]	<0.0001
PLR	21.03 [19.55]	12.95 [14.80]	<0.0001	<0.0001	<0.0001	28.33 [38.04]	26.97 [47.16]	0.1382

The hematological parameters, including WBCs, neutrophils, lymphocytes, monocytes, platelets, and their derived ratios (NLR, LMR, PLR) before (pre-) and after (post-) treatment for patients categorized by ICU admission are presented. Data are presented as median [IQR]. The *p*-values were calculated using the Wilcoxon matched-pairs signed rank test for within-group comparisons. *p* indicates the comparison between pre- and post-treatment within each group; *p* * compares pre-treatment values between the two groups; and *p* ^#^ compares post-treatment values between the two groups.

**Table 7:** Hematological parameters and NLR, LMR, and PLR values of patients based on length of hospital stay.

Variable	Length of Hospital Stay							
	<15 Days					≥15 Days		
	Pre-Treatment	Post-Treatment	*p*	p*	*p ^#^*	Pre-Treatment	Post-Treatment	*p*
WBCs (× 10^3^/μL)	14.89 [9.18]	11.01 [9.11]	<0.0001	<0.0001	<0.0001	12.67 [6.69]	7.74 [4.99]	<0.0001
Neutrophils (× 10^3^/μL)	84.20 [11.37]	80.80 [18.58]	<0.0001	<0.0001	<0.0001	79.40 [12.45]	69.00 [23.85]	<0.0001
Lymphocytes (× 10^3^/μL)	8.05 [8.38]	10.30 [13.55]	<0.0001	<0.0001	<0.0001	12.60 [0.30]	21.00 [19.80]	<0.0001
Monocytes (× 10^3^/μL)	4.50 [2.40]	4.90 [3.30]	0.0308	<0.0001	<0.0001	5.30 [2.75]	5.80 [2.55]	<0.0001
Platelets (× 10^3^/μL)	239.00 [126.70]	236.50 [141.00]	0.3393	0.2109	0.9028	235.00 [90.00]	240.00 [104.00]	0.0006
NLR	6.38 [5.88]	3.29 [4.98]	<0.0001	0.0002	<0.0001	7.28 [7.94]	3.98 [6.92]	<0.0001
LMR	1.78 [1.62]	2.15 [2.22]	<0.0001	<0.0001	<0.0001	2.35 [1.81]	3.49 [3.27]	<0.0001
PLR	29.11 [29.63]	20.53 [28.35]	<0.0001	<0.0001	<0.0001	19.27 [17.57].	12.00 [13.16]	<0.0001

The hematological parameters, including WBCs, neutrophils, lymphocytes, monocytes, platelets, and their derived ratios (NLR, LMR, PLR) before (pre-) and after (post-) treatment for patients based on the length of hospital stay are presented. Data are presented as median [IQR]. The *p*-values were calculated using the Wilcoxon matched-pairs signed rank test for within-group comparisons. *p* indicates the comparison between pre- and post-treatment within each group; *p* * compares pre-treatment values between the two groups; and *p* ^#^ compares post-treatment values between the two groups.

## Data Availability

If required, our data can be submitted.
